# Beyond Antioxidants: The Emerging Role of Nrf2 Activation in Amyotrophic Lateral Sclerosis (ALS)

**DOI:** 10.3390/ijms26209872

**Published:** 2025-10-10

**Authors:** Minoo Sharbafshaaer, Roberta Pepe, Rosaria Notariale, Fabrizio Canale, Gioacchino Tedeschi, Alessandro Tessitore, Paolo Bergamo, Francesca Trojsi

**Affiliations:** 1 First Division of Neurology and Neurophysiopathology, AOU Università degli Studi della Campania “Luigi Vanvitelli”, 80138 Naples, Italy; minoo.sharbafshaaer@unicampania.it (M.S.); notarialer@gmail.com (R.N.); fabrizio.canale@unicampania.it (F.C.); gioacchino.tedeschi@unicampania.it (G.T.); alessandro.tessitore@unicampania.it (A.T.); 2 Department of Advanced Medical and Surgical Sciences, University of Campania “Luigi Vanvitelli”, 80138 Naples, Italy; roberta.pepe@unicampania.it; 3 CNR-IBBR National Research Council, Institute of Biosciences and Bioresources, 80131 Naples, Italy; paolo.bergamo@cnr.it

**Keywords:** Nrf2, amyotrophic lateral sclerosis, RNA-binding proteins, oxidative stress, stress granules, mitochondrial dysfunction, nucleocytoplasmic transport, glial cells, neuroinflammation, Keap1 inhibitors

## Abstract

Amyotrophic lateral sclerosis (ALS) is a neurodegenerative disorder involving the progressive degeneration of upper and lower motor neurons. While oxidative stress, RNA-binding protein (RBP) pathology, mitochondrial dysfunction, and glial–neuronal dysregulation is involved in ALS pathogenesis, current therapies provide limited benefit, underscoring the need for multi-target disease-modifying strategies. Nuclear factor erythroid 2-related factor 2 (Nrf2), classically regarded as a master regulator of redox homeostasis, has recently emerged as a central integrator of cellular stress responses relevant to ALS. Beyond its canonical antioxidant function, Nrf2 regulates critical pathways involved in mitochondrial quality control, proteostasis, nucleocytoplasmic transport, RNA surveillance, and glial reactivity. Experimental models demonstrate that astrocyte-specific Nrf2 activation enhances glutathione metabolism, suppresses neuroinflammation, promotes stress granule disassembly, and reduces RBP aggregation. In *C9orf72*-linked ALS, Nrf2 activation mitigates dipeptide repeat protein toxicity and restores RNA processing fidelity via modulation of nonsense-mediated decay and R-loop resolution. Recent advances in Nrf2-targeted interventions including Keap1–Nrf2 protein–protein interaction inhibitors, dual Nrf2/HSF1 activators, and cell-type-selective Adeno-associated virus 9 (AAV9) vectors show promise in preclinical ALS models. These multimodal approaches highlight Nrf2’s therapeutic versatility and potential to address the upstream convergence points of ALS pathogenesis. Taken together, positioning Nrf2 as a systems-level regulator offers a novel framework for developing precision-based therapies in ALS. Integrating Nrf2 activation with RNA- and glia-directed strategies may enable comprehensive modulation of disease progression at its molecular roots.

## 1. Introduction: ALS and the Search for Multi-Target Neuroprotection

Amyotrophic lateral sclerosis (ALS) is a progressive, adult-onset neurodegenerative disorder characterized by the selective degeneration of both upper and lower motor neurons, leading to muscle weakness, spasticity, and atrophy. The disease almost invariably results in paralysis and respiratory failure within 3–5 years from symptom onset, with only ~10% of patients surviving beyond a decade [1]. Although most cases are sporadic, approximately 10% are familial, linked to pathogenic variants in genes, such as Superoxide Dismutase 1 (*SOD1*), Chromosome 9 open reading frame 72 (*C9orf72*), Fused in Sarcoma (*FUS*) and *TAR DNA-binding protein* (*TARDBP*) [2]. Pathological hallmarks include ubiquitinated cytoplasmic inclusions containing phosphorylated TAR DNA-binding protein 43 (pTDP-43) in >95% of cases, as well as less frequent aggregation of SOD1, FUS, or tau in select patient subsets [3,4].

Multiple, tightly interconnected pathogenic processes drive motor neuron loss in ALS. These include oxidative stress, mitochondrial dysfunction, impaired proteostasis, excitotoxicity, disrupted RNA metabolism, nucleocytoplasmic transport defects, and non-cell-autonomous toxicity mediated by reactive astrocytes and microglia [5,6,7,8,9]. Modern omics-based profiling and network analyses have reframed ALS not as a linear cascade of pathological events, but as a dynamic, self-reinforcing network in which disturbances in one domain (e.g., oxidative damage) rapidly propagate to others, including mitochondrial quality control, RNA-binding protein homeostasis, and neuroinflammatory signaling [10,11,12]. For example, pTDP-43 aggregation impairs RNA splicing and transport, disrupts mitochondrial bioenergetics, and triggers redox imbalance, which in turn accelerates further protein misfolding and aggregation [13,14]. Within this complex pathogenic landscape, Nrf2 should be viewed not as an isolated antioxidant factor but as a systems-level hub that interfaces with multiple dysregulated pathways. Through its regulation of oxidative stress, mitochondrial quality control, RNA metabolism, proteostasis, and neuroinflammatory responses, Nrf2 occupies a central position within the interconnected network that underlies ALS. This broader contextualization highlights that its therapeutic relevance stems from redox regulation and its capacity to integrate and rebalance several converging mechanisms that drive neurodegeneration.

The limited clinical efficacy of currently approved therapies against sporadic ALS—Riluzole, and Edaravone—highlights the inadequacy of approaches targeting single pathways in a multifactorial disease [15,16,17]. Riluzole, the first FDA-approved drug for ALS, primarily acts by stabilizing voltage-gated sodium channels in their inactivated state and reducing glutamate-mediated excitotoxicity, thereby decreasing neuronal hyperexcitability [18]. Edaravone, in contrast, functions mainly as a free radical scavenger that attenuates oxidative stress through neutralization of reactive oxygen species; recent studies further suggest additional protective effects on cellular metabolism and TDP-43 pathology in ALS-relevant neuronal models [19,20,21]. Despite their approval, both drugs confer only modest clinical benefit, reinforcing the need for therapeutic strategies capable of simultaneously modulating multiple converging pathogenic mechanisms, which may offer superior neuroprotection [22].

**Figure 1:**
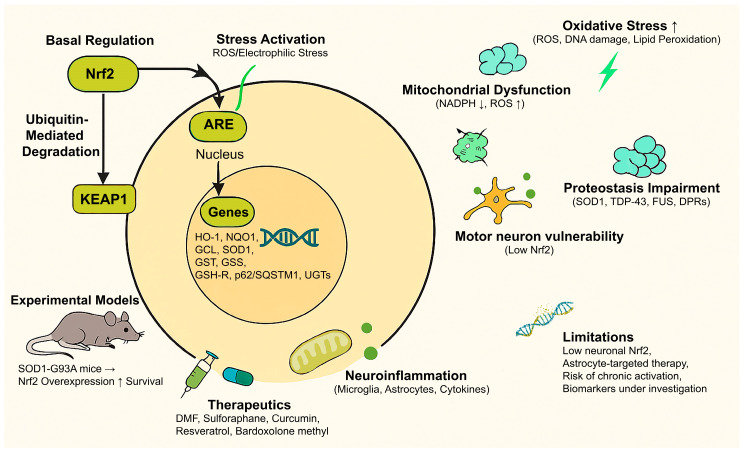
Under normal conditions, Nrf2 is bound to KEAP1 and degraded. Oxidative or electrophilic stress modifies KEAP1, allowing Nrf2 to enter the nucleus, bind to AREs, and activate genes that regulate antioxidant defense, detoxification, and proteostasis. In ALS, impaired Nrf2 signaling contributes to oxidative stress, mitochondrial dysfunction, protein aggregation, neuroinflammation, and motor neuron vulnerability. Experimental models show that Nrf2 activation delays disease onset, and therapeutic candidates such as DMF, sulforaphane, curcumin, resveratrol, and bardoxolone methyl are under investigation.

In this context, nuclear factor erythroid 2-related factor 2 (Nrf2) has emerged as a master regulator with unique potential to restore homeostasis across several domains of ALS pathobiology. Canonically, Nrf2 functions as the principal transcriptional activator of the antioxidant response via the Keap1–Nrf2–antioxidant response element (ARE) axis, regulating genes involved in glutathione synthesis, ROS detoxification, and xenobiotic metabolism [23]. However, recent work has expanded Nrf2’s repertoire to include regulation of mitochondrial biogenesis and mitophagy [24], lipid and energy metabolism [25], proteostasis via the ubiquitin–proteasome and autophagy–lysosome systems [26], anti-inflammatory signaling through suppression of NF-κB activity [27], and, notably, RNA metabolism and stress granule dynamics [28,29]. The fundamentals of Nrf2 in ALS pathogenesis are summarized in Table 1 and schematically illustrated in Figure 1, providing a conceptual framework for the following sections.

In ALS, Nrf2 expression and activity appear dysregulated in both neurons and glial cells. Post-mortem analyses reveal reduced nuclear localization of Nrf2 and diminished ARE-dependent transcription in spinal motor neurons of sporadic ALS patients, despite upregulation in reactive astrocytes [30,31]. Cell-type-specific studies show that Nrf2 activation in astrocytes reduces glutamate-mediated excitotoxicity and prolongs motor neuron survival, while in microglia, it suppresses pro-inflammatory cytokine release [32,33]. In *C9orf72*-ALS/FTD models, Nrf2 activation restores mitochondrial membrane potential, normalizes oxidative phosphorylation, and reduces dipeptide repeat protein-induced stress [34].

Importantly, the latest findings have revealed previously unappreciated RNA-centric roles for Nrf2. Transcriptomic profiling of Nrf2-activated ALS patient-derived motor neurons identified significant modulation of RNA-binding proteins implicated in ALS, including TDP-43, Heterogeneous nuclear ribonucleoprotein1 (hnRNPA1), and FUS, alongside altered expression of genes controlling nucleocytoplasmic transport and stress granule assembly [28,35]. Parallel proteomic studies suggest that Nrf2 may regulate the survival motor neuron (SMN) complex and spliceosome assembly factors, providing a potential mechanistic bridge between oxidative stress responses and RNA dysregulation [29,35]. These discoveries position Nrf2 not merely as an antioxidant transcription factor but as a multifaceted systems regulator capable of influencing the key molecular nodes where ALS pathogenetic mechanisms converge.

Preclinical data indicate that pharmacological or genetic activation of Nrf2, whether by electrophilic compounds such as dimethyl fumarate and nitro-fatty acids, dietary phytochemicals, like sulforaphane, or emerging targeted activators, can attenuate oxidative and proteotoxic stress, preserve mitochondrial integrity, modulate glial reactivity, and potentially restore RNA homeostasis in ALS models [34,35,36]. These multidimensional benefits suggest that therapeutics designed to harness or fine-tune Nrf2 signaling could serve as viable multi-target interventions for ALS, warranting translational exploration in precision medicine frameworks.

Therefore, this scoping review aims to critically synthesize current evidence on the multifaceted roles of Nrf2 in ALS pathogenesis, extending beyond its canonical antioxidant functions to encompass emerging regulatory mechanisms in mitochondrial dynamics, RNA metabolism, proteostasis, and glial–neuronal crosstalk. We further discuss recent advancements in Nrf2-targeted therapeutic strategies and propose a conceptual framework positioning Nrf2 as a central integrator of multi-target interventions in ALS.

## 2. Classical Nrf2 Pathway: Oxidative Stress and Redox Homeostasis

The imbalance between the formation of free radicals and the antioxidant capacity of the organism (generally known as oxidative stress) is usually associated with pathological conditions. Under physiological conditions, oxidant molecules are present in limited quantities in cells due to the presence of enzymatic and non-enzymatic antioxidants. Excessive production of free radicals or a deficit in the defence mechanism may be involved in the onset of ALS [37,38]. Among the regulators of the antioxidant response is Nrf2, a transcription factor that protects against various diseases by modulating intracellular antioxidant/detoxifying and anti-inflammatory responses [39]. The balance and stability of NRF2 are controlled by several adaptors, including Kelch-like ECH-associated protein 1 (KEAP1). KEAP1 is a cysteine-rich protein (27 cysteines) composed of 3 functional domains: the bric-à-brac (BTB) domain, an intermediate region (IVR), and a Kelch domain. Under physiological conditions, the Kelch domain of Keap1 interacts with the Neh2 domain of Nrf2 (containing the DLG and ETGE motifs), while the Bric-à-Brac (BTB) domain recruits the ubiquitin ligase Cullin3 for proteasomal degradation of Nrf2 [40]. Under oxidative stress, the conformation of KEAP1 changes, through the adduction or oxidation of cysteine residues, losing its normal function. Consequently, the KEAP1-Nrf2 dimer dissociates, and the accumulation and translocation of Nrf2 to the nucleus occur [41]. In the nucleus, Nrf2 binds to the regulatory DNA sequence of the antioxidant response element (ARE). The Nrf2/ARE complex is involved in the activation of genes for the synthesis of phase II enzymes, with antioxidant and detoxifying functions [42]. Among the genes containing ARE in the promoters are those encoding enzymes such as NAD(P)H quinone oxidoreductase 1 (*NQO1*), heme oxygenase-1 (HO-1), glutamate-cysteine ligase (GCL, catalytic subunit *GCLC* and regulatory subunit *GCLM*), glutathione synthetase (GS), glutathione peroxidase (*GPx*), glutathione reductase (*GSH-R*), thioredoxin (Trx), Trx Reductase (TrxR), peroxiredoxin (Pdx), and sulfiredoxin (Srxn) [43]. glutathione (GSH) is known for its antioxidant role in the central nervous system. In primary cultures of glia and neurons, overexpression of Nrf2 was associated with increased GSH synthesis, by increased glutathione synthase (GS) and consequent increased expression of glutathione S-transferase (GST) [44].

The detoxifying enzymes of greatest interest are heme HO-1, important in the neuroprotective process for the degradation products of the heme group, which have antioxidant activity [45], and NQO1 with antioxidant activity because it catalyzes the two-electron reduction of quinone to redox-stable hydroquinone, preventing the formation of free radicals [46].

Attention to Nrf2 has increased in recent years. Natural compounds and synthetic agents have been evaluated as potential activators of the Nrf2 pathway in the context of ALS. Among the most studied synthetic molecules, CPN-9 has shown selective neuroprotection against cell death due to oxidative stress, but with poor solubility and permeability of the blood–brain barrier [47]. WN1316 has provided remarkable results on motor function and survival in mutant SOD1 mice, but human data are limited [48]. Among natural compounds, Curcumin has recently been to improve mitochondrial dysfunction in NSC34 cells expressing the mutant variant of TDP43 [49]. In mutant SOD1 mice, treated with a Curcumin derivative (GT863), a slowing of the progression of motor dysfunction has been evaluated [50]. In humans, two preclinical studies have shown slowing of disease progression in ALS patients and reduction in oxidative damage [51]. Dimethyl fumarate, already used for the treatment of multiple sclerosis, is currently of particular interest since it is a molecule that activates the Nrf2 pathway and has an anti-inflammatory and antioxidant effect. In a phase 2 study conducted on patients diagnosed with sporadic ALS, good tolerability and safety were evaluated, but no significant improvement was found after 36 weeks [52]. A study in mice has shown that sulforaphane, a natural activator, taken with the diet reaches the brain and reduces neuroinflammation and oxidative stress. This molecule can regulate several biochemical pathways, such as alterations in redox homeostasis/immune response, including the Nrf2 pathway associated with neurodegenerative diseases [53]. Further studies are needed to analyze the impact of sulforaphane or dimethyl fumarate and their derivatives.

## 3. Astrocytic Nrf2 Activation: Glial Defence and Non-Cell Autonomous Protection

In recent years, increasing evidence has underlined that non-cell-autonomous mechanisms, where non-neuronal cells contribute to the progression of ALS, are crucial [54]. In particular, the astrocytes, a type of glial cell, play a crucial role in neuroprotection by modulating motor neuron survival through the activation of Nrf2 [45]. Several studies have demonstrated that the specific activation of Nrf2 in astrocytes can improve motoneuron survival in animal models of ALS [55]. Under physiological conditions, Nrf2 binds to the ARE region of DNA and stimulates the production of enzymes, such as HO-1, thioredoxin, and heat shock proteins, thereby contributing to the maintenance of redox balance in the brain [30,56,57]. Astrocytes, as the primary source of GSH in the brain, play a central role in antioxidant defence through the activation of the Nrf2 pathway [58,59]. They produce and transport GSH precursors to motoneurons, which is essential for neuronal protection by neutralizing free radicals and preventing oxidative damage [60]. The levels of GSH synthesis are higher in astrocytes compared to motoneurons, primarily due to the preferential activation of Nrf2-regulated genes encoding enzymes such as GCL and GS, which are crucial for GSH synthesis. This allows astrocytes to significantly increase GSH production and release in response to stimuli, creating an extracellular environment rich in this antioxidant [61,62].

Since motoneurons cannot efficiently synthesize or directly uptake GSH, they rely on astrocytes as a major source of antioxidant support. Astrocyte-derived GSH released into the extracellular space can directly neutralize reactive oxygen and nitrogen species (ROS and RNS), thereby providing a first line of defense against oxidative stress [59]. In addition, the presence of specific GSH-binding sites on the membranes of both astrocytes and neurons suggests a potential role as a glial-derived neuromodulator or neurotransmitter [63]. Most importantly, astrocytes supply motoneurons with GSH precursors, which are taken up and used to elevate intracellular GSH levels, thereby enhancing their capacity to counteract oxidative damage [64].

In models of ALS, Nrf2 activation in astrocytes predominantly occurs during advanced stages of degeneration as a compensatory protective response [65]. Notably, while motoneurons exhibit lower Nrf2 levels, glial cells such as astrocytes tend to express higher amounts of Nrf2, especially during the early stages of the disease, indicating a potential early protective role for these cells [66].

Although most of the evidence derives from in vitro models, recent in vivo studies have demonstrated that modifying Nrf2 activity in astrocytes can promote neuronal resistance to harmful stimuli. Transgenic overexpression of Nrf2 in astrocytes led to approximately a 25% increase in GSH content in the spinal cord and showed protective effects against toxicity induced by the SOD1 gene mutation, which is associated with ALS. In mouse models of ALS, as well as of Parkinson’s disease, and ischemia/ hypoperfusion, Nrf2 overexpression in astrocytes significantly reduced neuronal damage and improved overall brain function [67].

A crucial question concerns the mechanism of Nrf2 activation in response to low-level endogenous stress, such as mild oxidative stress, typical of pathological conditions and aging. Such stress activates Nrf2 through a Keap1-dependent mechanism: under resting conditions, the Kelch domain of Keap1 binds to the Neh2 domain of Nrf2, promoting its ubiquitin-dependent degradation and maintaining low cytoplasmic Nrf2 levels [36]. When oxidative stress occurs, redox-sensitive cysteines within Keap1 are modified, preventing this interaction. This allows newly synthesised Nrf2 to accumulate, translocate into the nucleus, and activate the protective gene program. This mechanism is crucial in the response to numerous neurodegenerative diseases, where the presence of dysfunctional Nrf2 pathway is associated with decreased neuroinflammation and oxidative stress [55,68].

The relevance of these findings is that pharmacological inducers of Nrf2, especially those acting through Keap1 inhibition, may remain effective even when oxidative stress is already active, complementing non-canonical activation pathways. This perspective suggests that Nrf2-based interventions could have therapeutic value not only at early but also at more advanced stages of ALS, when endogenous Nrf2 activity is already partially engaged [69]. Within this framework, enhancing Nrf2 activity in astrocytes is particularly promising, since selective modulation of this pathway can strengthen the natural defences of glial cells, limit oxidative and inflammatory injury, and in turn support the survival and function of motor neurons [70]. At the same time, moving these insights into clinical practice requires a deeper understanding of how Nrf2 operates through canonical and non-canonical mechanisms, across different cell types and at different disease stages. Such knowledge will be essential for the design of targeted interventions that fine-tune astrocytic Nrf2 activity, balance glial responses, and ultimately contribute to slowing disease progression and improving quality of life for people living with ALS [71].

It is crucial to develop approaches that activate Nrf2 in protective glial cells, such as astrocytes, while limiting their transformation into pro-inflammatory reactive forms [72]. Remarkably, during the pre-symptomatic stages of ALS, astrocytes may maintain a supportive role for motor neurons until a critical threshold; beyond this point, their protective capacity diminishes, and the disease manifests. Therefore, a therapeutic intervention focusing on Nrf2 could aim to strengthen glial defences, modulating inflammation and oxidative stress to create a more favourable environment for neuronal survival and slow disease progression.

## 4. Nrf2 and Mitochondrial Quality Control in ALS

Mitochondria are important organelles for the brain; neurons have high metabolic demands and require 20% of the ATP produced by mitochondria. Furthermore, these organelles modulate calcium metabolism and influence the release of neurotransmitters [73]. Neurons are long-lived cells and tend to accumulate cellular damage, including mitochondrial damage. Mitochondrial dysfunction has been associated with several neurodegenerative diseases, including ALS [8]. In particular, mitochondria with altered structure have been observed in motor neurons of ALS patients [74] and reported in studies on animal and cellular models [75]. Moreover, mitochondria play a role in maintaining cellular energy homeostasis. For this function, their morphology can change in response to alterations in metabolic status. Although mitochondria themselves produce ROS, they suffer several morphological modifications in the presence of oxidizing molecules [76]. When they suffer damage, the production of ROS increases, leading to the pathological condition of oxidative stress and the subsequent development of various pathologies [77]. Nrf2 influences mitochondrial function, in cooperation with other transcription factors, by inducing the synthesis of mitochondrial respiratory chain proteins [78]. Activation of the Nrf2 pathway in the presence of oxidant molecules protects mitochondria by stimulating mitophagy, an autophagy event that leads to the selective removal of only damaged mitochondria. PTEN-induced putative kinase 1 (*PINK1*) is a kinase located on the mitochondrial membrane that phosphorylates its substrates after cell damage. Specifically, phosphorylation of Parkin at Ser65 triggers its activation as an RBR E3 ubiquitin ligase, which adds ubiquitin to outer mitochondrial membrane proteins, depolarizing them [43]. The depolarization of membrane proteins is a recognition signal for autophagosomes that initiate the process of autophagy. Nrf2, in the presence of oxidant molecules, induces the expression of *PINK1*, which has four ARE sequences in the promoter [79]. Mitophagy can remove damaged mitochondria via p62 sequestosome 1 (p62), Keap1, and the E3 ubiquitin ligase RBX1. The *p62* gene also contains three ARE sequences in its promoter and is a target of the transcription factor Nrf2. The p62 protein also competes with Nrf2 for binding to the inhibitory protein Keap1 [24]. Furthermore, one of the two mitofusin genes, *MFN2*, contains ARE sequences in the promoter and responds to Nrf2 by increasing its expression [80]. Consequently, this regulation by Nrf2 promotes mitochondrial fusion, an event necessary for energy production and maintaining cellular health. Good perfusion is thought to protect against cell death. Nrf2 indirectly influences mitochondrial activity by modifying antioxidant levels, inducing the expression of mitochondrial proteins GSH-R, GPX, thioredoxin reductase 2, peroxiredoxin 3, peroxiredoxin 5, and Superoxide Dismutase 2 (SOD2), thus counteracting ROS accumulation [81]. For mitochondrial protection, several compounds that increase Nrf2 expression have been studied. Among these, previously mentioned, Sulforaphane in mice was able to preserve oxidative phosphorylation by inducing mitochondrial biogenesis and reducing ROS formation in the liver [82].

Mitochondrial dysfunction is a common feature of ALS/Fronto-temporal dementia (FTD) associated with G4C2 expansion in the *C9orf72* gene; however, it remains unclear whether this condition is a cause or a consequence of the pathological process [83]. Mitochondria are essential for cellular energy production (ATP), but during this process, they generate ROS, which can damage mitochondrial DNA and impair cellular function. In ALS, in particular, a decrease in Nrf2 activity due to reduced expression or altered regulation leads to a reduction in antioxidant defenses, resulting in increased oxidative stress, protein aggregation, and motor neuron death. Similarly, the *C9orf72* repeat expansion is associated with mitochondrial dysfunction and increased oxidative damage, contributing to disease progression [76].

Au et al. (2024) [34] analyzed multiple aspects of mitochondrial biology in various Drosophila models of C9orf72-ALS/FTD; these authors discovered that morphology, oxidative stress, and mitophagy are commonly altered, and these alterations correlate with a progressive loss of locomotor abilities. They highlighted that only genetic manipulations that reversed oxidative stress levels could recover motor deficits associated with *C9orf72* repeat expansion, thus supporting a causal link between mitochondrial dysfunction, oxidative stress, and behavioral phenotypes.

Therapeutic strategies focus on activating the Nrf2 pathway to restore the cellular antioxidant capacity. Several approaches are used.

Keap1 inhibitors such as sulforaphane and dimethyl fumarate (DMF) prevent Nrf2 degradation, allowing its translocation to the nucleus to promote the expression of antioxidant genes. However, despite activation, this has been demonstrated to be insufficient to prevent excess ROS and resulting neurotoxicity, which could be mitigated through transgenic overexpression of antioxidant enzymes [84]. From a translational perspective, it is encouraging that both genetic reduction in Keap1 and pharmacological targeting with DMF can suppress C9orf72 toxicity in vivo and in patient-derived neurons. In tests with the FDA-approved drug for ALS, Edaravone, DMF’s effects on reducing ROS were comparable to EDV’s, but at concentrations approximately an order of magnitude lower [34].

Other approaches include activation of p62 to sequester Keap1 and enhance Nrf2 activity; GSK-3β inhibitors and PI3K/AKT pathway activators to reduce Nrf2 degradation; targeting pathways such as 3-Hydroxy-3-methylglutaryl reductase degradation (HRD1) and BTB domain and CNC homolog 1 (Bach1) inhibitors to further promote Nrf2 activation; and anti-inflammatory agents that inhibit NF-κB to reduce neuroinflammation, which exacerbates oxidative stress [85,86].

Although some studies have reported Nrf2 pathway dysfunction in patient samples with ALS [55], data are very limited regarding *C9orf72*-linked ALS samples. Recently, Jiménez-Villegas et al. (2022) [87] demonstrated that Nrf2 activation is compromised in cell culture models of arginine dipeptide repeat protein (DPR) toxicity, where they also observed improved cell viability following DMF treatment.

We have expanded these findings by showing the benefit of targeting Keap1/Nrf2 in vivo and patient-derived neurons, reinforcing the importance of activating Nrf2 as a potential therapeutic target. A recent phase 2 clinical trial tested the efficacy of DMF in patients with sporadic ALS, with encouraging results [52]. Although the study concluded that there was no significant effect on the primary endpoint (i.e., Revised ALS Functional Rating Scale), a slowdown in neurophysiological decline was observed, suggesting preservation of lower motor neuron function. The authors also noted that participants exhibited an “unusually slow disease progression,” indicating the need for larger studies to confirm these findings.

In conclusion, enhancing Nrf2 efficacy represents a promising therapeutic strategy to counteract mitochondrial dysfunction, oxidative stress, and neurodegeneration in ALS, *C9orf72*-related diseases, and other neurodegenerative conditions. Further research is essential to optimize these strategies and better understand how these pathways are regulated so that effective treatments can be developed.

## 5. Nrf2 Meets RNA Metabolism: A New Frontier in ALS Mechanisms

Disruption of RNA metabolism is now recognized as a core neurophysiological feature of ALS, particularly in familial cases harboring mutations in *TARDBP* and *FUS* or repeat expansion in *C9orf72*. These alterations involve widespread deficits in RNA splicing, degradation, transport, and sequestration, often accompanied by pathological cytoplasmic aggregation of RNA-binding proteins (RBPs). In parallel, gain-of-function phenomena such as repeat-associated non-AUG (RAN) translation and persistent stress granule accumulation further exacerbate neuronal toxicity and disturb cellular excitability and axonal function. Recent studies have identified Nrf2 as a novel modulator of these RNA-driven mechanisms. Beyond its canonical antioxidant role, Nrf2 has recently been to regulate the expression of RBPs directly, promote stress granule disassembly, and restore nucleocytoplasmic transport in ALS-relevant neuronal and glial contexts [88].

In *C9orf72*-linked ALS models, activation of the Keap1–Nrf2 axis mitigates toxic RNA foci accumulation and DPR generation by enhancing RNA surveillance pathways, including nonsense-mediated decay and helicase-driven R-loop resolution [32,34,89]. In parallel, Nrf2 modulates redox-sensitive signaling cascades in astrocytes, rebalancing excitatory/inhibitory dynamics and protecting against synaptic failure through enhanced glial-neuronal crosstalk [32]. These mechanisms collectively reframe Nrf2 as a neurophysiological integrator, linking redox buffering, RNA metabolism, and proteostasis in ALS pathogenesis. This multifunctional regulatory profile supports Nrf2 as a mechanistically grounded and highly promising therapeutic target, particularly in strategies aimed at restoring molecular homeostasis and neuronal resilience early in the disease course.

### 5.1. Beyond Redox: Nrf2 as a Master Regulator of RNA-Binding Protein Homeostasis in ALS

Traditionally recognized for its role in maintaining redox homeostasis and regulating phase II detoxification enzymes via the canonical KEAP1–Nrf2–ARE signaling axis, Nrf2 has recently been implicated in the regulation of RBP dynamics in ALS. Emerging transcriptomic and proteomic data from ALS-relevant iPSC-derived astrocytes and motor neurons demonstrate that activation of Nrf2 leads to altered expression of key disease-associated RBPs, including hnRNPA1, FUS, and T-cell-restricted intracellular antigen 1, which are essential for mRNA splicing, axonal transport, stress granule dynamics, and RNA stability, respectively [90].

In ALS, RBPs frequently undergo pathological mislocalization and aggregation, processes often driven by disturbed phase separation and impaired proteostasis. Activation of Nrf2 appears to influence both transcriptional and post-transcriptional regulation of RBP-encoding genes, while also modulating protein turnover and localization through redox-sensitive signaling and autophagy-related mechanisms [91]. Specifically, Nrf2 enhances the expression of proteostasis-associated factors such as Heat Shock Protein 70 (HSP70), DNAJB6, and SQSTM1/p62 [92]. In *C9ORF72*, *SOD1*, *TARDBP*, *FUS*, and *NIMA Related Kinase 1* (*NEK1*), it upregulates RNA surveillance elements like UPF1 [93], hereby limiting cytoplasmic RBP aggregation under oxidative or proteotoxic stress. This body of evidence suggests that Nrf2 exerts a non-canonical yet crucial role in safeguarding RNA homeostasis by maintaining RBP integrity, broadening its impact beyond classical redox regulation, and reinforcing its promise as a therapeutic target in ALS and related proteinopathies.

### 5.2. Nrf2 in Stress Granule Clearance and Phase Separation Control

One of the most compelling recent discoveries is the role of Nrf2 in regulating stress granule (SG) dynamics and aberrant phase separation, both central to RNA metabolism and ALS pathogenesis. SGs are membrane-less, cytoplasmic condensates composed of untranslated mRNAs and RBPs, rapidly assembled under stress. While transient SGs formation serves a protective role, its pathological persistence, as observed in ALS, promotes RBP mislocalization, toxic aggregation, and disruption of RNA homeostasis [94].

Recent evidence indicates that Nrf2 activation enhances the expression of molecular chaperones (e.g., HSP70) and autophagy-related adaptors (e.g., p62/SQSTM1), supporting the disassembly and clearance of persistent stress granules (SGs) and misfolded RBPs through selective autophagy [93]. Nrf2 also influences the levels and redox sensitivity of phase-separation regulators such as G3BP1 (protein), which control the physical state of SGs and their transitions between liquid-like and solid-like assemblies [95]. By restoring healthy phase-separation dynamics, Nrf2 not only prevents SGs from maturing into pathological inclusions but also ties its antioxidant activity to broader protein quality control. In this light, Nrf2 emerges as both a buffer against oxidative stress and a guardian of proteostasis, positioning it at the crossroads of stress responses, RNA granule biology, and neurodegeneration, and positioning it as a viable therapeutic axis in ALS.

### 5.3. Nrf2 Crosstalk with Nucleocytoplasmic Transport and RNA Surveillance

Disruption of nucleocytoplasmic transport is a hallmark of ALS pathology, contributing to the nuclear depletion and cytoplasmic accumulation of RBPs such as TDP-43 and FUS. Evidence suggests that Nrf2 activation can mitigate these abnormalities by upregulating key nuclear transport factors, including karyopherin subunit alpha-2 (KPNA2) and exportin-1 (XPO1), and stabilizing their redox-sensitive cysteine-rich domains, which are otherwise vulnerable to oxidative modification [96,97]. Through this mechanism, Nrf2 facilitates proper nucleocytoplasmic shuttling of RBPs and maintains nuclear transport fidelity under stress conditions.

In parallel, Nrf2 has recently been to influence multiple aspects of RNA surveillance, including the nonsense-mediated mRNA decay (NMD) pathway, R-loop resolution, and RNA helicase regulation. These pathways are essential for eliminating aberrant transcripts and resolving RNA–DNA hybrids that can interfere with transcription and genomic integrity [98,99,100]. In the context of *C9orf72*-associated ALS/FTD, where expanded GGGGCC (G4C2) repeats form nuclear RNA foci and produce toxic DPRs via repeat-associated non-AUG (RAN) translation, Nrf2 activation has been reported to reduce both RNA foci and DPR accumulation [34,101]. This effect is likely mediated through the combined enhancement of RNA quality control systems and proteasomal degradation pathways. Through these actions, Nrf2 emerges as a multifaceted safeguard of RNA and nucleocytoplasmic homeostasis, two interdependent systems that are critically impaired in ALS pathogenesis.

### 5.4. Therapeutic Outlook: Integrating Nrf2 Activation into RNA-Targeted Strategies

The convergence of Nrf2 signaling with ALS-relevant RNA regulatory pathways opens new avenues for therapeutic intervention. Unlike antisense oligonucleotides (ASOs) or siRNAs, which selectively target individual transcripts, Nrf2 activation offers a systems-level strategy that modulates multiple intersecting domains of ALS pathogenesis, including oxidative stress, RBP dynamics, SG behavior, nucleocytoplasmic transport, and RNA surveillance [40,102,103].

Emerging preclinical models support the rationale for combination approaches. For example, Adeno-associated virus 9 (AAV9)-mediated delivery of astrocyte-specific Nrf2 vectors or blood–brain barrier (BBB)-permeable KEAP1 inhibitors may restore redox and RNA homeostasis in non-neuronal cells, thus may restore the non-cell-autonomous dysfunction that precedes overt neurodegeneration in ALS [66,69]. These interventions could be synergistically combined with RNA-directed therapeutics such as ASOs targeting C9orf72 transcripts or DPRs to enhance both neuronal survival and glial support.

Recent discoveries reframe Nrf2 as a pivotal molecular hub in ALS pathogenesis, with roles extending far beyond its canonical redox-responsive transcriptional activity. Current evidence indicates that Nrf2 orchestrates not only antioxidant defense and mitochondrial function but also exerts direct transcriptional and post-transcriptional regulation over critical RNA processing pathways, including RBP homeostasis, SG clearance, and nucleocytoplasmic RNA trafficking [66,102,104]. In C9orf72-linked ALS/FTD, Nrf2 activation alleviates key molecular pathologies by reducing DPR accumulation and RNA foci, restoring nuclear TDP-43, and enhancing RNA quality control pathways such as NMD and R-loop resolution. These effects target the core RNA metabolism defects characteristic of *C9orf72* disease and align with broader RNA-based therapeutic strategies aimed at restoring transcriptome integrity [69,90,105,106].

Taken together, multiple strategies for activating Nrf2, through pharmacological agents, natural compounds, or genetic and epigenetic modulation act on interconnected pathways that include antioxidant defense, mitochondrial integrity, RNA metabolism, and neuroinflammation. These approaches consistently demonstrate protective effects in experimental models, but their translation to meaningful clinical benefit remains limited. This gap underscores the need for therapeutic strategies that integrate Nrf2 activation with complementary interventions to more effectively address the multifactorial nature of ALS.

## 6. Pharmacological Activation of Nrf2: From Repurposed Drugs to Novel Compounds

Given the expanding evidence for Nrf2 as a central regulator of oxidative stress, mitochondrial integrity, RNA metabolism, proteostasis, and neuroinflammation, pharmacological modulation of this pathway has emerged as a promising strategy in ALS. Unlike monotherapeutic approaches that target isolated molecular lesions, Nrf2 activation offers a systems-level intervention, addressing multiple converging pathogenic processes that contribute to both neuronal and non-neuronal dysfunction.

In the context of ALS marked by molecular heterogeneity, interplay between redox imbalance and RBP misregulation, and widespread nucleocytoplasmic disruption, Nrf2 activation holds appeal as a unifying therapeutic axis [69,93,102]. Preclinical and early translational studies have demonstrated that boosting Nrf2 activity can simultaneously attenuate oxidative damage, enhance autophagic clearance of stress granules, promote RNA quality control, and support mitochondrial dynamics, especially in astrocyte–neuron crosstalk models.

This section provides an overview of the pharmacological landscape of Nrf2 activation, encompassing

- Repurposed FDA-approved agents, such as DMF, Sulforaphane, and Curcumin, which exert partial Nrf2 induction through modification of KEAP1 cysteine residues or activation of upstream kinases.
- Second-generation Nrf2 activators, including bardoxolone methyl and omaveloxolone, are designed for improved potency, bioavailability, and BBB penetration.
- Dual-pathway compounds, such as hybrid molecules targeting both Nrf2 and NF-κB or Nrf2 and autophagy pathways, are potentially able to fine-tune the neuroinflammatory and proteostatic environment in ALS.
- Gene therapy and delivery strategies, including AAV-mediated Nrf2 expression and astrocyte-specific enhancers, offer targeted modulation with minimal off-target effects.

As drug discovery efforts advance, prioritizing brain-permeable, cell-type-specific, and chronically safe Nrf2 modulators will be critical for translating these findings into effective, disease-modifying therapies for ALS.

### 6.1. Repurposed Clinical Drugs with Nrf2-Activating Properties

Several clinically approved pharmacological agents initially developed for non-neurological applications have demonstrated Nrf2-activating capabilities through diverse molecular mechanisms. These include covalent modification of reactive cysteine residues on Keap1, leading to disruption of Keap1–Nrf2 complex formation, as well as indirect activation via redox-sensitive kinase signaling cascades such as MAPK, PKC, and PI3K/AKT, which converge on Nrf2 phosphorylation and nuclear stabilization [107,108].

The translational appeal of these repurposed compounds lies in their well-characterized pharmacokinetic, safety, and tolerability profiles in humans. In ALS, a disease marked by prolonged development timelines and high clinical trial attrition, such agents represent an accelerated route to clinical application, particularly when their mechanisms align with disease-relevant targets, including oxidative stress, mitochondrial dysfunction, RNA dysregulation, and glial pathology [108,109].

#### 6.1.1. Dimethyl Fumarate (DMF)

DMF is an orally available electrophilic compound approved for the treatment of multiple sclerosis and psoriasis. It has emerged as one of the most extensively characterized Nrf2-inducing agents in the context of neuroinflammation and neurodegeneration. DMF acts by alkylating key thiol residues on Keap1, particularly cysteine 151, leading to a conformational disruption of the Keap1–Nrf2 complex. This prevents Nrf2 ubiquitination and degradation, allowing its nuclear translocation and activation of ARE-driven transcriptional programs [108,110].

In ALS-relevant *SOD1* and *TDP-43* transgenic models, DMF has demonstrated a range of neuroprotective effects:

- Upregulation of Nrf2 target genes (*HMOX1*, *NQO1*, *GCLC*), enhancing glutathione synthesis and antioxidant buffering.
- Suppression of microglial activation and downregulation of NF-κB-mediated proinflammatory signaling, reducing neuroinflammatory burden.
- Improvement in mitochondrial bioenergetics, including increased membrane potential, ATP production, and Ca^2+^ buffering capacity in astrocytes and motor neurons.
- Delays in disease onset and prolonged survival in ALS mouse models, indicating disease-modifying potential [110,111].

Importantly, DMF has recently been to influence protein homeostasis mechanisms. It enhances autophagy–lysosomal flux and proteasome activity, contributing to the clearance of misfolded proteins and persistent stress granules, hallmark features of ALS pathology [111]. A study using iPSC-derived ALS astrocytes further demonstrated that DMF treatment reduced oxidative stress markers such as 4-hydroxynonenal (4-HNE) and malondialdehyde (MDA), while improving astrocyte-mediated synaptic support, supporting its role as a multifunctional neuroprotective agent [112]. These findings position DMF as a clinically relevant compound capable of targeting multiple ALS-related pathomechanisms via Nrf2 activation, with translational value supported by existing human safety data and ongoing formulation development for central nervous system (CNS) indications.

#### 6.1.2. Sulforaphane (SFN)

SFN is a naturally occurring isothiocyanate derived from glucoraphanin, abundant in cruciferous vegetables such as broccoli and Brussels sprouts. It acts as a potent indirect activator of Nrf2, primarily via covalent modification of redox-sensitive cysteine residues on Keap1, most notably Cys151, leading to conformational destabilization of the Keap1–Nrf2 complex. This inhibits proteasomal degradation of Nrf2 and promotes its nuclear translocation and transcriptional activation of ARE-driven cytoprotective genes [113,114].

In ALS-relevant models, particularly using iPSC-derived astrocytes and co-culture systems, SFN has demonstrated multimodal neuroprotective effects:

- Reduces endoplasmic reticulum (ER) stress and enhances the unfolded protein response (UPR), contributing to proteostasis restoration.
- Upregulates phase II detoxifying enzymes, including glutathione S-transferases (GSTs), UDP-glucuronosyltransferases (UGTs), and thioredoxin reductase 1 (TXNRD1), thereby improving redox buffering capacity [115].
- Restores glutathione homeostasis and prevents redox-mediated astrocyte dysfunction, ultimately preserving neuronal viability in neuron–astrocyte co-cultures under oxidative stress [116].
- Suppresses inflammatory gene expression via inhibiting NF-κB signaling and modulation of reactive glial phenotypes.

While SFN’s broad cytoprotective actions position it as a promising therapeutic candidate, its short plasma half-life, rapid phase II metabolism, and limited BBB permeability represent significant pharmacokinetic barriers to CNS efficacy. To address these limitations, novel delivery platforms, including nanoencapsulation, liposomal formulations, and SFN-loaded polymeric micelles, are under investigation to enhance bioavailability, stability, and brain targeting [117]. Altogether, SFN provides a strong proof of concept for dietary-derived Nrf2 activation in ALS, though clinical translation will depend on overcoming pharmacological limitations through innovative formulation strategies.

#### 6.1.3. Curcumin

Curcumin, a pleiotropic polyphenol derived from Curcuma longa, has gained renewed interest in neurodegenerative research due to its ability to modulate redox-sensitive signaling pathways, neuroinflammation, mitochondrial function, and protein homeostasis. Mechanistically, Curcumin activates Nrf2 via ROS-mediated oxidation of Keap1 cysteine thiols, disrupting the Keap1–Nrf2 complex and facilitating Nrf2 nuclear translocation. It enhances ARE-driven transcription via upstream kinases, including JNK, ERK1/2, and PI3K/AKT, thus promoting a broad cytoprotective gene program [66,118].

Recent ALS-focused studies have revealed that curcumin exerts multiple neuroprotective effects:

- Inhibits NF-κB and NLRP3 inflammasome activation, reducing neuroinflammatory cascades in motor neurons and glial cells [119,120].
- Upregulates Nrf2 target genes, such as *HMOX1*, *NQO1*, and *FTH1*, promoting ROS detoxification and iron homeostasis [121].
- Reduces protein aggregation, including misfolded SOD1 and C-terminal TDP-43 fragments in ALS spinal cord tissue [121].
- Improves mitochondrial function, restoring membrane potential and ATP production in ALS-relevant cellular models [49].

Despite its mechanistic appeal, Curcumin’s clinical translation is limited by poor oral bioavailability, rapid hepatic metabolism, and low BBB permeability. To overcome these issues, novel analog (e.g., EF24, CDF) and advanced formulations (e.g., liposomal, nanoparticle-based, and polymeric delivery systems) are under preclinical development to enhance CNS penetration and pharmacodynamic stability [109,122]. Altogether, curcumin represents a multi-target Nrf2 modulator capable of influencing several ALS-relevant pathways, though further pharmacological optimization is required for therapeutic viability in human trials.

### 6.2. Novel Synthetic Compounds and Targeted Nrf2 Activators

#### 6.2.1. Non-Electrophilic Keap1–Nrf2 Protein–Protein Interaction (PPI) Inhibitors

Advancements in structure-based drug design have enabled the development of non-electrophilic small molecules that selectively disrupt the Keap1–Nrf2 protein–protein interaction (PPI), offering enhanced specificity and safety over traditional electrophilic Nrf2 activators. These compounds bind the Kelch domain of Keap1, competitively blocking its interaction with the ETGE and DLG motifs of Nrf2’s Neh2 domain, thus stabilizing cytosolic Nrf2 and promoting its nuclear accumulation without the need for reactive cysteine modification [123].

Unlike classical Nrf2 activators (e.g., DMF, sulforaphane), these PPI inhibitors avoid broad redox reactivity, resulting in lower off-target toxicity, improved pharmacokinetics, and enhanced CNS penetration features that are critical for translational utility in neurodegenerative diseases. Preclinical studies have demonstrated that brain-permeable Keap1–Nrf2 PPI disruptors confer neuroprotection in ALS mouse models by:

- Reducing neuroinflammation through microglial reprogramming.
- Preserving motor neuron survival.
- Improving functional outcomes and prolonging lifespan [91].

This approach represents a shift from general oxidative stress modulation to targeted Nrf2 pathway reactivation through precise molecular interference, providing a promising pharmacological scaffold for ALS drug development.

#### 6.2.2. M102: A Dual Nrf2–HSF1 Pathway Modulator

M102 is a next-generation dual-pathway small molecule that simultaneously activates Nrf2 and heat shock factor 1 (HSF1), two major transcriptional regulators of cellular homeostasis. By co-inducing antioxidant defenses and proteostasis machinery, M102 addresses key converging pathomechanisms in ALS, including oxidative damage, protein misfolding, and mitochondrial dysfunction [124].

Mechanistically, M102 enhances

- Nrf2-dependent transcription of cytoprotective genes.
- HSF1-driven expression of molecular chaperones such as HSP70, HSP40, and HSPB8, essential for disaggregation and clearance of toxic protein aggregates.

In ALS models, including zebrafish expressing mutant *TDP-43* and rodent *SOD1G93A* lines M102 has recently been used to improve motor neuron viability, to reduce cytoplasmic stress granule burden, to restore mitochondrial membrane potential and bioenergetic function, and to delay neuromuscular degeneration and extend functional lifespan [124]. This dual-targeted strategy exemplifies a systems-level therapeutic approach, simultaneously reinforcing two complementary axes of neuroprotection. As a prototype, M102 supports the rationale for combinatorial transcription factor targeting in ALS and other proteinopathy-driven disorders.

### 6.3. RNA-Targeted Nrf2 Interventions

The expanding understanding of Nrf2 as a modulator of RBP homeostasis, stress granule dynamics, and nucleocytoplasmic RNA trafficking has catalyzed the development of RNA-centered pharmacological strategies that intersect with redox signaling. These advances aim to exploit the non-canonical roles of Nrf2 in the regulation of post-transcriptional RNA metabolism, particularly relevant in ALS, where mislocalization and aggregation of RBPs (e.g., TDP-43, FUS) are central to pathogenesis [125].

Recent pharmacological innovations involve dual-function Keap1–Nrf2 antagonists engineered simultaneously. Their main activities are to stabilize Nrf2, enhancing expression of antioxidant and cytoprotective genes (e.g., *HMOX1*, *NQO1*), and to regulate nucleocytoplasmic transport, specifically by modulating levels and function of KPNA2 (importin-α1) and XPO1 (exportin-1), thereby restoring proper localization of nuclear RBPs [100,125,126]. In ALS-relevant cellular systems, these compounds have been shown to restore nuclear TDP-43 and FUS localization, reversing pathological cytoplasmic aggregation; to augment antioxidant gene expression, reinforcing redox homeostasis; and to promote clearance of aberrant RNA granules and RBP inclusions, alleviating proteotoxic stress [125,126,127].

Crucially, when paired with ASOs such as those targeting pathogenic C9orf72 G4C2 repeats or mutant *FUS* transcripts, these dual-function Nrf2 modulators yield synergistic therapeutic effects, including reduction in RNA foci and DPR burden, enhancement of RNA surveillance pathways, including NMD and R-loop resolution, and restoration of nucleocytoplasmic transport fidelity and proteostasis [125,126,127]. By integrating these complementary mechanisms, RNA-centric Nrf2 targeting emerges as a precision therapeutic platform, combining transcriptomic rescue, proteostatic correction, and oxidative stress modulation with broad implications for both familial and sporadic ALS.

### 6.4. Delivery Strategies and Cell-Type Specific Modulation

Despite the therapeutic potential of Nrf2 activation in ALS, a major obstacle remains the poor CNS bioavailability of most Nrf2-targeting compounds. Recent innovations in drug delivery and genetic engineering have sought to overcome these limitations through cell-type-specific strategies and enhanced BBB penetration.

Key approaches include

- CNS-targeted nanoparticles encapsulating Nrf2 agonists with hydrophilic or labile properties. These nanoparticles are often coated with BBB-penetrating ligands (e.g., transferrin, RVG peptides), enabling efficient delivery of small molecules or siRNA to motor neurons and glial cells [128,129].
- AAV9-based gene therapy platforms engineered to drive astrocyte-specific Nrf2 expression using glial fibrillary acidic protein (GFAP) promoters. In *SOD1G93A* ALS mouse models, systemic delivery of AAV9-GFAP-Nrf2 constructs:
- Delayed disease onset.
- Preserved motor neuron count in spinal cord regions.
- Prolonged survival compared to controls [45].

Keap1-silencing RNA therapeutics, including synthetic miRNAs, shRNAs, or CRISPR–Cas9 systems, are delivered either via viral vectors or lipid-based carriers. These agents promote endogenous Nrf2 activation in astrocytes and microglia, bolstering antioxidant defenses and mitigating non-cell-autonomous toxicity [45,128].

The next-generation wave of Nrf2-targeted interventions, combining cell-type selectivity with transcriptional precision. Importantly, these delivery innovations enable targeting of glial dysfunction, a central and early driver of ALS progression, without perturbing neuronal redox balance inappropriately. As our understanding of Nrf2’s multifaceted role in RNA homeostasis, mitochondrial regulation, protein aggregation, and glial–neuronal crosstalk deepens, the therapeutic paradigm will likely shift toward combinatorial strategies. These may integrate Nrf2 activation with antisense oligonucleotides, RNA-binding protein modulators, or mitochondrial enhancers, paving the way for mechanism-driven, disease-modifying therapies in ALS and related neurodegenerative disorders.

## 7. Conclusions

Over the past decade, the conceptualization of ALS has progressed from a motor neuron-centric pathology to a complex multisystem neurodegenerative disorder encompassing oxidative stress, mitochondrial dysfunction, dysregulated RNA metabolism, proteostasis collapse, and glial–neuronal uncoupling. Within this expanded pathogenic framework, Nrf2 has emerged as a master regulatory node with the capacity to orchestrate protective transcriptional responses across multiple converging disease pathways, positioning it as a compelling therapeutic target in ALS [36,130].

Traditionally recognized for orchestrating antioxidant defence via the transcriptional activation of cytoprotective genes (e.g., *HMOX1*, *NQO1*, *GCLC*), Nrf2 is now understood to modulate a broader spectrum of cellular processes directly implicated in ALS pathogenesis [41,131,132]. These include the following.

- Regulation of RBPs: Nrf2 influences the phase separation dynamics of RBPs such as TDP-43 and FUS, whose aggregation into persistent cytoplasmic inclusions is a hallmark of ALS [125,133].
- Control of nucleocytoplasmic transport: Emerging evidence indicates that Nrf2 activation can restore the balance of importin/exportin-dependent transport mechanisms (e.g., via KPNA2 and XPO1), rescuing nuclear TDP-43 localization and ameliorating RBP dysfunction [125,134].
- Stress granule clearance and proteostasis maintenance: through its downstream effects on autophagy–lysosomal flux and proteasomal activity, Nrf2 facilitates the removal of misfolded proteins and stress granules, thus preserving proteome integrity in motor neurons [30,135].
- Mitochondrial quality control is a key interface between oxidative stress, neurodegeneration, and ferroptosis regulation. Nrf2 activation promotes mitochondrial biogenesis, membrane potential stability, and calcium buffering, functions impaired in ALS astrocytes and motor neurons [136,137]. As mitochondrial dysfunction can influence ferroptotic susceptibility, degradation pathways play dual roles depending on whether they target anti- or pro-ferroptotic modulators. Selective modulation of these pathways could offer therapeutic potential in neurodegenerative diseases.

These pleiotropic effects position Nrf2 as a systems-level integrator capable of modulating the upstream determinants of ALS progression, rather than merely buffering downstream oxidative injury. Recent studies also implicate Nrf2 in regulating ferroptosis, UPR, and ER–mitochondrial signaling, further enhancing its relevance in neurodegeneration [31,90,138].

Despite its therapeutic potential, current understanding of Nrf2 largely stems from in vitro and acute rodent studies, with minimal validation in genetically diverse ALS models or patient-derived organoids. Importantly, Nrf2 activation by DMF and 4-octyl itaconate promotes Annexin A1 secretion through the cholesterol transporter ABCA1, revealing a key anti-inflammatory mechanism that should be investigated in more representative disease models [139]. Second, the spatiotemporal dynamics and cell-type specificity of Nrf2 signalling remain poorly defined. It is unclear whether sustained activation across diverse glial and neuronal populations confers synergistic neuroprotection or risks inducing transcriptional imbalance and metabolic stress. Evidence from multiple sclerosis models demonstrates that DMF can activate Nrf2 in neurons, astrocytes, and oligodendrocytes, leading to preserved myelin, axons, and neuronal integrity through antioxidant and anti-inflammatory pathways. However, these broad cellular effects highlight the need to determine how Nrf2 activation should be temporally and spatially regulated to maximize therapeutic benefit while minimizing potential adverse consequences [108].

The pharmacokinetics and CNS penetrance of many small-molecule Nrf2 activators are suboptimal. Repurposed agents such as dimethyl fumarate and Sulforaphane, an isothiocyanate derived from Brassica vegetables, exhibit neuroprotective effects across various models of neurodegeneration, primarily via activation of the Nrf2/ARE pathway. While its safety profile is favourable, suboptimal BBB penetration may limit its therapeutic potential in ALS [140]. Notably, recent preclinical work demonstrated that intranasal delivery of sulforaphane via iron oxide nanoparticles enhanced its neuroprotective efficacy, mitigating oxidative stress, preserving neuromuscular function, and improving histopathological outcomes in cisplatin-induced neurotoxicity—highlighting nanoparticle-based delivery as a potential strategy to overcome pharmacokinetic limitations [141].

Advanced delivery systems, including CNS-targeted nanoparticles and liposomal encapsulations, are being actively explored to enhance the brain penetration and cellular specificity of Nrf2 activators. For example, electrophilic neurite outgrowth-promoting prostaglandin compounds selectively accumulate in neurons, bind to Keap1 in a thiol-dependent manner, and activate the Nrf2/HO-1 pathway, thereby protecting against oxidative stress and excitotoxicity. Such targeted delivery strategies may improve therapeutic efficacy while minimizing off-target effects in neurodegenerative diseases [142]. Meanwhile, recognizing the pivotal role of astrocytes in oxidative stress regulation and neuroinflammatory control, recent preclinical studies have demonstrated that precision-targeted gene therapies using AAV9 vectors coupled with astrocyte-specific promoters (e.g., GFAP) can selectively enhance Nrf2 activity in these cells, resulting in prolonged survival and preserved motor neuron integrity in ALS models [143].

In parallel, novel classes of Keap1–Nrf2 PPI inhibitors and dual-pathway modulators such as M102 (Nrf2/HSF-1 activator) demonstrate superior brain penetrance and broader mechanistic coverage by simultaneously targeting redox signaling, proteostasis, and stress granule dynamics [124].

Overall, these advancements support the fundamental role of Nrf2 as a potential central node in the ALS therapeutic network. By modulating key processes at the intersection of RNA surveillance, mitochondrial homeostasis, and proteostasis, Nrf2 activation may enable a shift toward upstream, disease-modifying interventions. The future trajectory of Nrf2-based therapeutics will likely require combinatorial strategies integrating pharmacological activators, gene therapies, and ASOs, tailored to specific ALS subtypes and progression phenotypes. Importantly, the development of robust pharmacodynamic biomarkers of Nrf2 activation and pathway engagement will be essential for translating these approaches into clinical success. Ultimately, harnessing the multifaceted regulatory potential of Nrf2 offers a promising avenue to address the mechanistic complexity of ALS and pave the way for more effective, precision-guided neuroprotective therapies.

Despite these promising perspectives, several limitations must be acknowledged. First, the real translational potential of Nrf2 activation in ALS remains uncertain, as most supporting evidence derives from preclinical or short-term models, with limited validation in large, genetically diverse patient cohorts. Second, the complexity and heterogeneity of ALS may reduce the efficacy of a single-axis therapeutic approach, even one as multifaceted as Nrf2 activation. Finally, chronic or sustained Nrf2 stimulation could entail potential risks, including metabolic imbalance, impaired redox signaling, or even oncogenic effects, which remain largely unexplored in long-term clinical settings. Future research should therefore focus not only on optimizing delivery strategies and biomarkers of pathway engagement but also on systematically assessing the safety and durability of Nrf2-based therapies across disease stages and patient subgroups.

## Figures and Tables

**Table 1:** Fundamentals of Nrf2 in ALS Pathogenesis.

Category	Description
Nrf2 Overview	Nrf2 is a transcription factor that regulates the expression of antioxidants and cytoprotective genes via the ARE pathway.
Basal Regulation	In unstressed conditions, Nrf2 is bound to KEAP1 in the cytoplasm and targeted for ubiquitin-mediated degradation.
Stress Activation	Upon oxidative/electrophilic stress, KEAP1 is modified, allowing newly synthesized Nrf2 proteins to a) escape Keap1-mediated ubiquitination b) translocate to the nucleus, c) bind to AREs, and activate gene transcription.
Main Target Genes	E.g., encoding *HO-1*, *NQO1*, *GCL*, *SOD1*, *GST*, *GSS*, *GSH-R*, *p62/SQSTM1*, proteasomal subunits, *UGTs*, involved in antioxidant defense, detoxification, and proteostasis.
Oxidative Stress in ALS	Elevated ROS, lipid peroxidation, and oxidative DNA/protein damage are prominent in both familial and sporadic ALS.
Nrf2 Role in Redox Balance	Induces antioxidant enzymes and glutathione biosynthesis pathways; reduces ROS accumulation.
Mitochondrial Dysfunction	ALS shows impaired mitochondrial function; Nrf2 supports mitochondrial health through NADPH production and mitochondrial gene regulation.
Proteostasis Impairment	Misfolded protein aggregation (SOD1, TDP-43, FUS, and DPRs in C9orf72-ALS); Nrf2 enhances autophagy and UPS via p62/SQSTM1 to promote aggregate clearance.
Neuroinflammation	Microglial and astrocytic activation drive neurodegeneration; Nrf2 suppresses inflammatory cytokines and supports anti-inflammatory glial phenotypes.
Motor Neuron Vulnerability	Motor neurons have intrinsically low Nrf2 levels; glial-specific Nrf2 activation provides non-cell-autonomous protection.
Experimental Models	In ALS models (e.g., SOD1-G93A mice), Nrf2 overexpression delays onset and prolongs survival; astrocyte-targeted Nrf2 shows stronger effects.
Therapeutic Candidates	DMF, Sulforaphane, Curcumin, Resveratrol, and Bardoxolone Methyl, preclinical evidence supports Nrf2 activation as a therapeutic strategy.
Limitations & Gaps	Neuronal Nrf2 expression is low; targeted delivery to astrocytes may be needed. Long-term activation may carry metabolic or oncogenic risks.
Clinical Translation	Early-phase trials with Nrf2 activators are ongoing; biomarkers of Nrf2 activation in ALS patients are still under investigation.

Abbreviation: ALS, Amyotrophic Lateral Sclerosis; ARE, Antioxidant Response Element; DMF, Dimethyl Fumarate; DPR, dipeptide repeat protein; FUS, Fused in Sarcoma; *GCL*, Glutamate–Cysteine Ligase; *GSS*, Glutathione Synthetase; *GSH-R*, Glutathione reductase; *GST*, Glutathione S-Transferase; *HO-1*, Heme Oxygenase-1; KEAP1, Kelch-like ECH-Associated Protein 1; NADPH, Nicotinamide Adenine Dinucleotide Phosphate; *NQO1*, NAD(P)H Quinone Dehydrogenase 1; Nrf2, Nuclear Factor Erythroid 2-Related Factor 2; *p62/SQSTM1*, Sequestosome-1; ROS, Reactive Oxygen Species; *SOD1*, Superoxide Dismutase 1; TDP-43, TAR DNA-Binding Protein 43; *UGTs*, Uridine 5′-Diphospho-Glucuronosyltransferase; UPS, Ubiquitin–Proteasome System.

## Data Availability

No new data were created or analyzed in this study. Data sharing is not applicable to this article.
